# A Review of Metabolomic Profiling in Rheumatoid Arthritis: Bringing New Insights in Disease Pathogenesis, Treatment and Comorbidities

**DOI:** 10.3390/metabo12050394

**Published:** 2022-04-27

**Authors:** Bárbara Jonson Bartikoski, Marianne Schrader de Oliveira, Rafaela Cavalheiro do Espírito Santo, Leonardo Peterson dos Santos, Natália Garcia dos Santos, Ricardo Machado Xavier

**Affiliations:** 1 Laboratório de Doenças Autoimunes, Universidade Federal do Rio Grande do Sul (UFRGS), Porto Alegre 90035-903, RS, Brazil; barbarabartikoski@gmail.com (B.J.B.); m.schraderdeoliveira@gmail.com (M.S.d.O.); rcsanto@hcpa.edu.br (R.C.d.E.S.); leopetersondossantos@gmail.com (L.P.d.S.); nataliagarcia.bio@gmail.com (N.G.d.S.); 2 Serviço de Reumatologia, Hospital de Clínicas de Porto Alegre (HCPA), Porto Alegre 90035-903, RS, Brazil; 3 Postgraduate Program in Medical Science, Universidade Federal do Rio Grande do Sul, Ramiro Barcelos 2400, Porto Alegre 90035-003, RS, Brazil; 4 Postgraduate Program in Biological Sciences: Pharmacology and Therapeutics, Barcelos 2400, Porto Alegre 90035-003, RS, Brazil

**Keywords:** rheumatoid arthritis, metabolomics, biomarkers, muscle wasting

## Abstract

Metabolomic analysis provides a wealth of information that can be predictive of distinctive phenotypes of pathogenic processes and has been applied to better understand disease development. Rheumatoid arthritis (RA) is an autoimmune disease with the establishment of chronic synovial inflammation that affects joints and peripheral tissues such as skeletal muscle and bone. There is a lack of useful disease biomarkers to track disease activity, drug response and follow-up in RA. In this review, we describe potential metabolic biomarkers that might be helpful in the study of RA pathogenesis, drug response and risk of comorbidities. TMAO (choline and trimethylamine oxide) and TCA (tricarboxylic acid) cycle products have been suggested to modulate metabolic profiles during the early stages of RA and are present systemically, which is a relevant characteristic for biomarkers. Moreover, the analysis of lipids such as cholesterol, FFAs and PUFAs may provide important information before disease onset to predict disease activity and treatment response. Regarding therapeutics, TNF inhibitors may increase the levels of tryptophan, valine, lysine, creatinine and alanine, whereas JAK/STAT inhibitors may modulate exclusively fatty acids. These observations indicate that different disease modifying antirheumatic drugs have specific metabolic profiles and can reveal differences between responders and non-responders. In terms of comorbidities, physical impairment represented by higher fatigue scores and muscle wasting has been associated with an increase in urea cycle, FFAs, tocopherols and BCAAs. In conclusion, synovial fluid, blood and urine samples from RA patients seem to provide critical information about the metabolic profile related to drug response, disease activity and comorbidities.

## 1. Introduction

Rheumatoid arthritis (RA) is the most common chronic, inflammatory autoimmune disease, which affects approximately 1% of the world’s population [1]. RA is characterized by joint inflammation with pain and swelling, which can lead to irreversible cartilage and bone damage. The etiology is still unknown, but environmental and genetic factors are involved with the disease’s susceptibility and severity [2]. The pathophysiology of RA results from the activation of self-reactive T and B cells, which lead to synovitis, cellular infiltration and a disorganized process of bone destruction and remodeling. The joint space is lined by a synovial membrane that suffers a tumor-like enlargement, called pannus, with local destructive effects [1,3,4].

In addition to joint involvement, RA patients often present metabolic disorders, such as insulin resistance and dyslipidemia contributing to an increased risk of cardiovascular problems and mortality [5]. Among complications presented by RA patients changes in body composition, such as loss of lean mass, especially skeletal muscle mass, and/or increase in fat mass, reduced muscle strength and poor physical function contributes to low patient survival and disability [6,7,8]. These changes are commonly reported in RA patients; however, methods for assessing muscle loss in clinical practice are limited, can be expensive and are not widely available [7,9].

Metabolic changes presented by RA patients can be linked to pathogenic mechanisms and may represent a possible way to understand environmental and genetic factors related to inflammatory diseases [10]. Since metabolism is important to the regulation of immune cells development, immunometabolism studies bring new insights about pathogenicity mechanisms, drug response, management of comorbidities and disease follow-up [5,11,12,13]. Metabolic products of several biological routes in the human body consist of diverse structures such as ionic chemical compounds, hydrophilic inorganic species, carbohydrates, volatile alcohols, ketones, organic acids, amino acids, lipids and complex natural products [14]. Thus, this review summarizes the current evidence about potential metabolic biomarkers in RA and their association with several clinical aspects of the disease.

## 2. Metabolomics

Metabolomic was introduced by Roger Williams in the late 1940s with the concept that individuals may have a “metabolic profile” reflecting the composition of metabolites of their biological fluids, consisting of a great variety of chemical structures with distinct functional properties [15]. The advent of metabolomics has been fueled by major improvements in instrument technology such as mass range of mass spectrometry (MS), associated gas, liquid chromatography, and laser-induced fluorescence (LIF). As a result, metabolomic patterns began to be associated with illnesses such as schizophrenia, inflammatory bowel disease (IBD) and cardiovascular diseases (CVD) [13]. Unlike transcriptome and proteomics, the molecular identification of metabolites cannot be deduced from genomic information. Global profiles of the genome, transcripts and proteins are based on the chemical analysis of target sequences composed of 4 different nucleotides (genome and transcriptome) or 22 amino acids (proteome), which are chemically similar facilitating the high throughput analytical approach [16]. The methodologies employed in metabolomics depend upon a training data set in which the outcome (i.e., disease or health) is known and used to build a predictive model; after training, the model may be used on a test set to classify unknown samples and measure the model’s predictive accuracy. The optimal selection of metabolites depends on the objectives of the study and is usually a compromise between sensitivity and selectivity of the technique chosen [17].

### 2.1. Techniques Used in Metabolomics

There are several types of metabolomics experiments including both targeted and untargeted analyses available for researchers according to the experiment’s purpose. Targeted analyses focus on identifying and quantifying a limited number of metabolites with good repeatability and stability [7]. On the other hand, the untargeted metabolomics approach focuses on the simultaneous detection of unknown specimens for a wide range of the detection of metabolite features with diverse chemical/physical properties. The main idea of untargeted approach is to quantify a large list of small molecules and to map the networks involved. While both targeted and untargeted approaches have their pros and cons, the challenge for the researcher is to maximize the detection and accurate identification of metabolites with a decent detection dynamic range and quantification capability [18].

The detection and separation of analytes are crucial for a successful metabolomic analysis. The separation analysis often used consists in liquid chromatography (LC), gas chromatography (GC) and capillary electrophoresis (CE), while for detection, MS, and nuclear magnetic resonance (NMR) methods are used for metabolomics experiments.

NMR methods are characterized by its nondestructive aspect and higher selectively, which means that the samples can be recovered and remeasured [19]. NMR consists in detecting characteristic radio frequency absorption bands (resonances) that occur as a result of placing molecules or molecular mixtures in very strong magnetic fields. The strong magnetic fields reorient the nuclear spins in each atom and make them susceptible to radio frequency excitation/absorption at very specific frequencies or chemical shifts. Each molecule has a unique pattern of NMR chemical shifts due to the unique chemical structure and distinct arrangement of hydrogen atoms around the molecule. These hydrogen chemical shift fingerprints allow compounds to be identified and quantified by NMR without the need for chromatographic separation or molecular ionization [16]. Differently, MS technology (including LC-MS (Liquid chromatography–mass spectrometry), GC-MS (Gas chromatography–mass spectrometry), CE-MS (Capillary electrophoresis–mass spectrometry) and IMS-MS (Ion mobility spectrometry–mass spectrometry)) is 10–100× more sensitive than NMR and a good combination of selectivity and sensitivity [20]. For all MS-based techniques, the target molecule suffers ionization for its detection and posterior identification [21]. The process gives neutral molecules either a positive or a negative charge (depending on the character of the molecule), and it is possible to measure the mass-to-charge (m/z) ratio of the ionized molecules, or their ionized molecular fragments; by comparing this information with other referential MS spectra of known compounds, it is possible to determine the identity of a given compound. However, the ionization process vaporizes the sample, so MS is an inherently destructive technique [20].

Nevertheless, modern NMR and MS instruments are capable of separating, detecting and characterizing hundreds to thousands of chemicals in complex chemical mixtures, such as biofluids or tissue extracts [20]. In most metabolomic studies, the instruments (NMR or MS) produce spectra or chromatograms consisting of thousands of peaks, which correspond to one or more unique compounds (in MS) or part of a single compound (in NMR). Because most unique compounds have overlapping peaks, large databases containing referential MS or NMR spectra of pure compounds must be used to determine which peaks in these spectra matches to certain chemical compounds [20,21,22].

In addition to the complex methodology available and integrative datasets for metabolomics, the metabolic profile of biological specimens is affected by numerous external factors such as diet, age, ethnicity, drugs, lifestyle or gut microbiota populations, and these factors need to be either controlled or deconvoluted to obtain information specific to health and disease [23].

### 2.2. Metabolic Profile in Health and Disease

Most intracellular metabolites produced are involved in the regulation of several biochemical reactions, which together constitute the cellular metabolic signaling network that is important in the cell regulation of growth, differentiation and death [23]. Metabolome analysis is capable of qualitatively and quantitatively detecting the profile of all low-molecular-mass metabolites (molecular mass less than 1000 Daltons–Da) present in cells and/or secretions that participate in metabolic reactions and intermediate compounds. Metabolic pathways can interact and overlap in a manner where ~3000–5000 metabolites may be detected from a single sample. There are endogenous metabolites such as lipids, small peptides, amino acids, organic acids, vitamins, carbohydrates, thiols and nucleic acids; or from an exogenous source as drugs, environmental contaminants, food additives, toxins and other xenobiotics [23,24]. Since metabolites are products of cell systems, many conditions are associated with a decrease/increase in particular metabolites, in a way that metabolic routes and their products are widely investigated to establish a link between the disease and metabolic profiles [24,25,26,27,28,29,30,31]. Choline and trimethylamine oxides (TMAOs), products of gut metabolism, have been implicated in studies due to their relation with CVD and metabolic modulation (Table 1) [32,33,34,35,36,37,38,39,40]. Furthermore, increases in branched-chain amino acids (BCAA’s) are observed the inflammatory state and are capable of promoting the increase in pro-inflammatory cytokines such as IL-6 and TNF-α [41,42,43]. On the other hand, the lack of key metabolites may present a serious repercussion on health, such as low levels of glutamine, which is known as “the fuel of metabolism” and has been implicated as a major source of energy in cells of the immune system [44,45,46,47]. Disturbances in glutamine metabolism are related to tumor growth, cardiovascular problems and metabolic syndrome [27,45]. A list of the main metabolites involved in health/disease status is described in Table 1.

**Figure 1:**
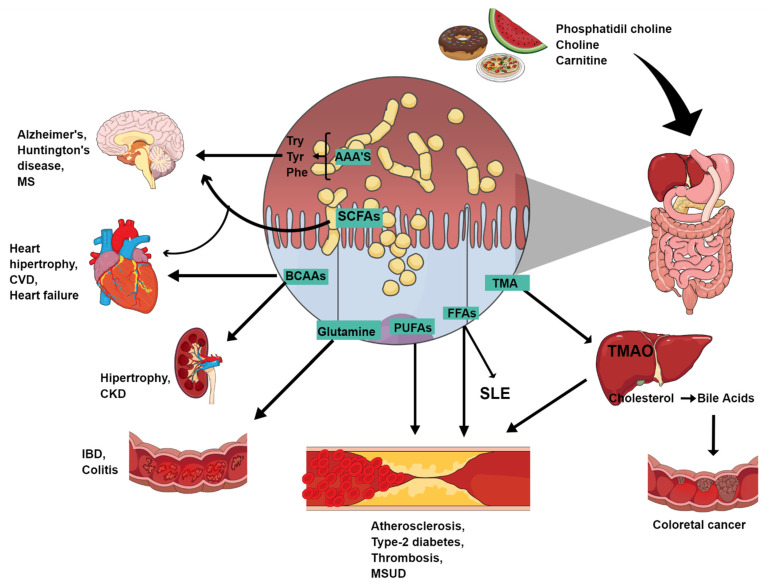
Potential role and mechanisms of action of gut microbiota metabolites in disease development. Abbreviations: SLE: systemic lupus erythematosus; CKD: chronic kidney disease; MS: multiple sclerosis; TCA: tricarboxylic acid; CRC: colorectal cancer; MSUD: maple syrup urine disease; IBD: inflammatory bowel disease; CVD: cardiovascular diseases.

Recent studies have shown that microbial metabolites are emerging effectors mediating the role of microbiota on host immune responses and its interactions [60]. Gut microbial metabolites contain a variety of molecules ranging from short-chain fatty acids (SCFAs) and vitamins to secondary bile acids and neurotransmitters, which act locally in the intestine and remotely exert their diverse effects on other organs [61]. SCFAs have been verified to participate in the regulation of innate immunity and antigen-specific adaptive immunity [39,51,52,53]; they can promote the generation of peripheral regulatory T-cells and suppress macrophage activation in an inflammatory response [62,63,64]. Therefore, many immune-related inflammatory disorders, such as IBDs, diabetes mellitus, RA and systemic lupus erythematosus (SLE), have been associated with altered gut microbiota [63,65,66]. In SLE, bile acids, including deoxycholic acid, GCA, isohyodeoxycholic acid and arachidonic acid, were significantly correlated with the SLEDAI score [54,67]. Increased levels of primary bile acids, including cholic acid (CA), glycocholic acid (GCA), taurocholic acid (TCA) and glycochenodeoxycholic acid (GCDCA), were also observed in feces from SLE patients, and they showed power to predict the SLEDAI score [28,50,58,59]. RA, which systematically affects the joints, has also been associated with microbiota-derived metabolites, which may be potential targets for the treatment of RA due to modulatory effects (Figure 1) [68,69,70].

## 3. Metabolic Profile in RA

**Figure 2:**
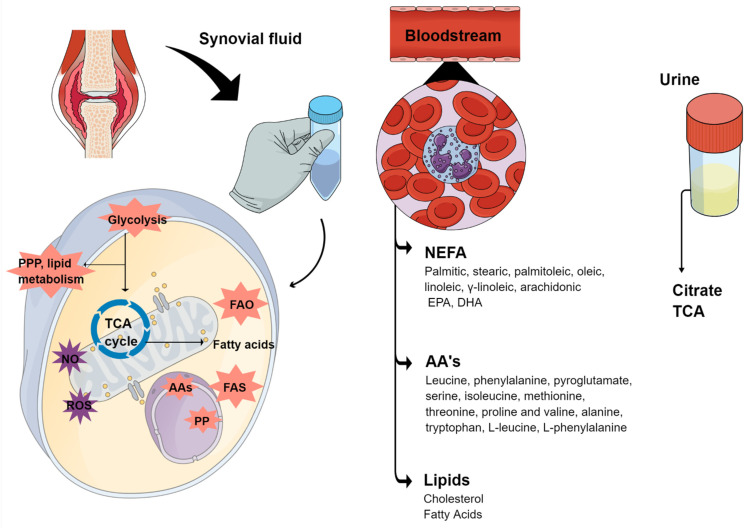
Metabolites commonly found in RA samples involved in the disease pathogenesis. Abbreviations: PPP: pentose phosphate pathway; TCA: tricarboxylic acid cycle; PP: pirimidine and purine; AAs: aminoacids; ROS: reactive species of oxygen; NO: nitric oxide; FAO: fatty acid oxidation; FAS: fatty acid synthesis; NEFA: nonesterified Fatty Acids; EPA: eicosapentaenoic acid; DHA: docosahexaenoic acid.

Metabolomics has been applied for a better understanding of the metabolic profile in RA patients, which suffers from a lack of diagnostic and prognostic markers [71]. RA pathogenesis includes synovial hyperplasia and pannus formation, which consists of an accumulation of macrophages and fibroblast-like synoviocytes (FLS) in the joint, which results in enhanced invasiveness and the destruction of cartilage and bone [72]. The aggressive phenotype of FLS can also be characterized by reduced contact inhibition, resistance to apoptosis, increased migration and increased ability to invade periarticular tissues [73]. These activated cells produce several mediators that induce angiogenesis, cell growth and the activation of immune cells contributing to the inflammatory environment and the production of local matrix metalloproteases (MMPs) that degrade the extracellular matrix and contribute to cartilage destruction [74]. Lately, several studies have shown that FLS activation and subsequent joint damage are associated with an altered metabolism represented by changes in four major classes of macromolecules: carbohydrates, proteins, lipids and nucleic acids (Figure 2). Due to the disrupted metabolism, several studies start to use synovial fluids from RA patients as a therapeutically target; 130 proteins were found to be uniquely present in synovial fluid and not observed in circulation (serum or plasma), which can be associated with metabolic changes and the joint environment [75]. Carlson et al. (2021) reported 30 metabolites such as putative RA biomarkers including various phospholipids, diol and its derivatives; arsonoacetate, oleananoic acid acetate; docosahexaenoic acid methyl ester; and linolenic acid and eicosatrienoic acid derivatives. Correspondingly, Wang et al. (2021) suggested disturbed pyrimidine metabolism, purine metabolism, fatty acid biosynthesis and unsaturated fatty acid biosynthesis, as well as increased naringenin levels, which are characteristics of the metabolism of RA [76].

As the final response to disease pathogenesis, metabolites may complement and indicate how RA patients respond to the environment, nutrition, disease progression, infections, exposure to xenobiotic agents, pharmacological treatment and other influences. Metabolomic studies of body fluids described that immune-mediated inflammatory diseases such as RA are associated with metabolic disruption, particularly for an increase in bioenergetic and biosynthetic demands to sustain chronic inflammation in the damaged tissue [77].

Comparing serum, urine and synovial fluids of healthy individuals and RA patients, the metabolic products of TCA, Free Fatty Acids (FFA), polyunsaturated fatty acids (PUFA), Prostaglandins, Thromboxanes and Leukotrienes, SCFA and Bile Acids (BA) are commonly increased in RA (Table 2) [40,48,49,50,54,55,56,57]. Moroever, the combined concentration parameter calculated as [aspartic acid] + [threonine] + [tryptophan] − [histidine] − [phenylalanine] presented a strong association with pain joint count, swollen joint count and DAS [78,79].

### 3.1. Lipidomic Profile in AR

Lipidomics is a large-scale study of pathways of cellular lipids in biological systems and is a subset of metabolome studies. During the last decade, lipids have attracted considerable attention as their role in the development, and the follow-up of diseases became increasingly recognized, particularly because lipid species such as prostaglandins (PG) and lipid mediators play crucial roles in the tight regulation of inflammation by acting as signaling molecules in the production of cytokines and chemokines [80]. Recent studies have investigated lipid mediators generated by cyclooxygenases or lipoxygenases during the preclinical stage of RA as well as the role of PG, including PGE2, and leukotrienes, such as LTB4, in the onset and development of arthritic diseases [1,81,82,83]. Therefore, changes in lipid mediators can be observed before disease manifestation [82,83]. For example, 5-hydroxyeicosatetraenoic acid (5-HETE) is elevated in the preclinical phase, while ω-3 fatty acid levels decreased in pre-RA subjects and short-chain carnitine levels decreased in serum samples obtained prior to RA onset [84,85].

**Figure 3:**
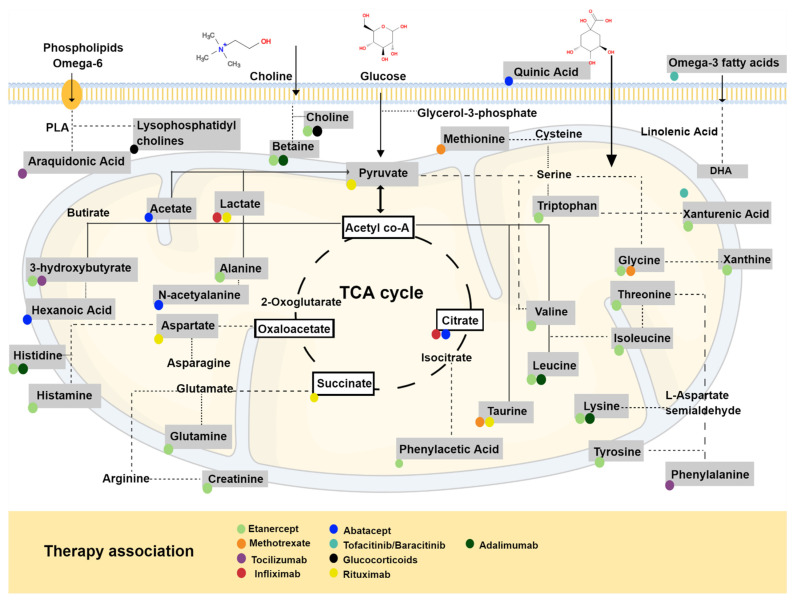
RA therapeutics influence in the metabolic profile. Abbreviations: TCA: tricarboxylic acid; PLA: phospholipase A; DHA: docosahexanoic acid.

In RA, synovial fluid-altered lysophosphatidylcholine/phosphatidylcholine ratios, as well as higher amounts of cholesterol, cholesterol esters and changes in the phospholipid composition, have been reported (Figure 3) [86,87,88,89,90].

Seventy different lipid components from distinct lipid classes in synovial fluid have been identified and lipoxin A4 and resolvin D5 and 5S,12S-diHETE are major markers of lipoxygenase pathway interactions in the samples of RA patients [91]. Some lipids perturbations may be seen in the in the serum of active RA and be distinct from sera of sustained remission patients; moreover, a global lipidomic analysis showed that subclasses of lysophosphatidylcholine, phosphatidylcholine, ether-linked phosphatidylethanolamine and sphingomyelin were correlated with RA activity and reflected treatment responses to anti-rheumatic drugs when monitored serially and could be potential biomarker to predict the evolution of preclinical to clinical disease stages [92].

Thus, lipidome analysis in RA could facilitate the assessment of disease activity and treatment outcomes allowing a more accurate analysis when combined with key metabolites of patient body fluids.

### 3.2. Metabolites in the Synovial Fluid

The joint is one of the major sites of inflammation in RA; it is lined with a thin, soft-tissue membrane called the synovial membrane that ensures the structural integrity of a normally organized synovial lining and the resident cells secretes hyaluronic acid and lubricin, two important constituents of synovial fluid that are responsible for lubricating the joint [93,94,95]. There are two types of resident cells in the synovial membrane: (1) FLS: mesenchymal-derived cells, which assemble fibroblasts that are known by UDP-glucose 6-dehydrogenase expression and CD55, a complement decay-accelerating factor [96]; and (2) synovial tissue macrophages (STM): a mixed population of local prenatal cells and those differentiated from circulating monocytes [97]. STM and FLS from the synovium are capable of changing their behavior toward the production of enzymes, which are responsible for cartilage and bone destruction in RA [95]. During the development of the disease, FLS and STM have metabolic changes; they shift from aerobic oxidative phosphorylation to a glycolytic state as the inflammation progresses, in which less adenosine triphosphate (ATP) is produced per cycle, but at a faster rate, to be able to meet energy requirements needed by highly active cells. Metabolites related to the glycolytic pathway, such as succinate, lactate and itaconate, have been detected in several studies in animal models, as well as in human metabolomics studies [98,99,100,101,102,103,104] (Table 2). 

**Table 2:** Metabolites associated with RA pathogenesis.

Sample	Sample Size (n)	Method Applied	Outcome	Reference
Synovial Fluid	48	GC/TOF MS	Positive correlation with DAS-28ES: radipate, fucose, glycocyamine, indole-3-lactate, isothreonate, phenylalanine and tryptophan asparagine Negative correlation with DAS-28ESR: citrate, cyano-/-alanine, oxoproline and ơ-alanine	[81]
Synovial Fluid	38	GC/TOF MS	Succinate, octadecanol, asparagine, terephthalate, salicylaldehyde, glutamine, citrulline, tyrosine, uracil, lysine, ribitol, tryptophan, xylose, ribose, isopalmitic acid, glycerol, myristic acid, palmitoleic acid, hydroxylamine and ethanolamine were validated as putative biomarkers for RA and discriminated from non-RA diseases	[84]
Synovial Fluid	3	LC-MS	Upregulated in RA: ibuprofen metabolism, glucocorticoid and mineralocorticoid metabolism, alpha-linolenic acid metabolism and steroid hormone biosynthesis. Downregulated in RA: purine and pyrimidine metabolism, arginine and proline metabolism; citrulline-nitric oxide cycle and glutathione metabolism.	[48,73]
Synovial Fluid	20	LC-MS	Activation of pyrimidine metabolism and purine metabolism, suppression of fatty acid biosynthesis and unsaturated fatty acid biosynthesis in RA	[105]
Blood	25	GC-MS	Decrease in histidine and threonic acid, methionine, asparagine, cholesterol in RA patients; Increase in glyceric acid, D-ribofuranose and hypoxanthine in RA patients	[82]
Plasma	47	1H NMR spectroscopy	Cholesterol, lactate, acetylated glycoprotein, and lipid signatures were found to be possible biomarkers for disease severity	[85]
Plasma	64	UPLC-MS/MS	Acylcarnitine metabolites are increase in lower disease activity. Glucuronate and hypoxanthin were found to be significantly increased in higher disease activity	[95]
Plasma	20	GC-MS	L-cysteine, citric acid and L-glutamine	[92]
Serum	53	indirect calorimetry	Increases in metabolic rate in RA patients smokers compared to non-smokers patients	[83]
Serum	27	GC/TOF MS and UPLC−QTOF MS	Increases in homoserine, 4,8-dimethylnonanoyl carnitine, glyceraldehyde, lactic acid, dihydroxyfumaric acid and aspartic acid are shared between 4 types of arthritis	[87]
Serum	58	Spectrophotometer	RA patients presented methyl-histidine and hydroxyisocaproic acid, while hexose-phosphate and fructose-6-phosphate distinguished high ADA from low ADA	[94]
Serum	124	LC-MS/MS	Serum levels of NEFA (palmitic, stearic, palmitoleic, oleic, linoleic, γ-linoleic, AA, linolenic, EPA and docosahexaenoic–DHA). The NEFA profile in RA patients is associated with clinical characteristics of aggressive disease and enhanced Th1 response.	[88]
Serum	33	GC-MS	Disturbances of leucine, phenylalanine, pyroglutamate, serine, isoleucine, methionine, threonine, proline and valine), fatty acids (palmitelaidate, oleate, trans-9-octadecenoate, cis-5,8,11-eicosatrienoate, docosahexaenoate, 2-ketoisocaproate and 3-methyl-2-oxovalerate) and carbohydrates (mannose, ribose, scyllo-inositol, glycerol and 1,5-anhydrosorbitol)	[89]
Serum	20	1H-NMR	Valine, isoleucine, lactate, alanine, creatinine, GPC APC and histidine relative levels were lower in RA, whereas 3-hydroxyisobutyrate, acetate, NAC, acetoacetate and acetone relative levels were higher compared with healthy controls.	[90]
Serum	30	LC-MS	4-methoxyphenylacetic acid, glutamic acid, L-leucine, L-phenylalanine, L-tryptophan, L-proline, glyceraldehyde and fumaric acid are possible biomarkers for RA	[91]
Serum and urine	Serum (*n* = 126) and urine (*n* = 83)	NMR	Increased glycolysis, perturbation in the citrate cycle, oxidative stress, protein catabolism and increased urea cycle activity are present in newly presenting RA patients with elevated CRP.	[93]
Urine	1400	1H-NMR	Lower levels of citrate were found in urine samples on RA patients	[86]

Abbreviations: UPLC−QTOF MS: liquid chromatography quadrupole-time-of-flight mass spectrometry; GC/TOF MS: gas chromatography/time-of-flight mass spectrometry; BCAA: branched-chain amino acids; 1H NMR: UPLC-Q-TOF-MS: ultra-performance liquid chromatography coupled with quadrupole time-of-flight mass spectrometry; 1H-NMR: one-dimensional (1-D) 1H spectra nuclear magnetic resonance; NEFA: nonesterified Fatty Acids; EPA: eicosapentaenoic acid; AA: arachdonic Acid; DHA: docosahexaenoic acid.

Yang et al. (2015) described the synovial fluid in RA patients with a decrease in isoleucine, valine, methionine, threonine, alanine and histidine levels, which could be a disturbance in glycolysis, TCA cycle, amino acids and lipid metabolism [100]. In addition, a couple of studies described the metabolome profile of FLS with profound metabolic differences in a late stage of RA when compared with osteoarthritis (OA) [106]. In FLS, an increased expression of nicotinamide phosphoribosyltransferase (NAMPT), which maintains adenine dinucleotide and nicotinamide (NAD) levels under stress conditions, was found in RA patients and mice with collagen-induced arthritis [107,108,109].

Another report showed that platelet-derived growth factor (PDGF) increases glucose metabolism and glucose transporter 1 expression in synoviocytes, and by inhibiting glucose metabolism, there is a decrease in inflammatory cytokine secretion, proliferation and migration of FLS [110]. Choline, responsible for catalyzing the phosphatidylcholine biosynthesis, is highly expressed in RA synovium and is induced by stimulation with TNF or PDGF [111]. The inhibition of choline kinase represses the aggressive behavior of rheumatoid FLS and attenuates disease in mice with serum transfer arthritis [112].

Lactate is the end product of glycolysis, a metabolic pathway that is upregulated in FLS and activated macrophages. The high concentrations of lactic acid are found in the blood and synovial fluids of inflamed joints in patients with RA. Studies have shown that lactate promotes the aggressive phenotype of FLS, the pro-inflammatory properties of macrophages stimulate IL-17 secretion by CD4+ T cells and at the same time decreases CD4+ T migration, which is related to the maintenance of a chronic inflammatory infiltrate [113]. The TCA cycle is widely studied in RA pathogenesis, including products such as succinate that activate NLRP3 inflammasome-inducing IL-1β secretion by synovial fibroblasts in a mouse model of RA and Itaconate, a TCA metabolite present high levels in an animal model of RA [114,115].

Despite the advances in synovial metabolic profile, the joint requires invasive techniques, which is uncomfortable for patients. Thus, blood and urine from patients with RA are often used in research to associate clinical parameters with the systemic effects of the disease.

### 3.3. Metabolites in Blood, Plasma and Serum of RA Patients

Lipids, amino acids and carbohydrates are the most abundant metabolites in plasma and blood. Blood samples are recognized as a good resource to obtain information about the disease status in RA patients [102]. Madsen et al., 2011 found a strong association with compounds such as glyceric acid, D-ribofuranose and hypoxanthine and a positive association with higher rates of nucleotide synthesis in the serum of RA patients; however, these data remain speculative [116]. Surowicec et al. (2016) demonstrated that meaningful disturbances in metabolic pathways might be involved in pre-symptomatic individuals’ years before the onset of RA [115]. Later, similar studies identified decreased levels of amino acids and glucose; increased levels of fatty acids and cholesterol, which were primarily associated with glycolytic pathway, fatty acid and amino acid metabolism; and other related pathways including TCA and urea cycles (Table 2) [103,117,118]. In this sense, it was found that serum metabolites are differentially expressed in rheumatic diseases from healthy controls constituting a unique metabolic signature of each type of arthritis, which may be used as biomarkers for diagnosis and patient stratification [119,120]. Among the metabolites identified, homoserine, 4,8-dimethylnonanoyl carnitine, glyceraldehyde, lactic acid, dihydroxyfumaric acid and aspartic acid were possible candidates as biomarkers shared by four types of arthritis (rheumatoid arthritis, osteoarthritis, ankylosing arthritis and gout) compared with healthy controls [120] (Table 2). In RA, adenosine deaminase (ADA) has been reported as a potential biomarker since patients presented different patterns of metabolic enzymes according to ADA concentration [121]. Thus, 4-methoxyphenylacetic acid, glutamate, L-leucine, L-phenylalanine, L-tryptophan, L-proline, glyceraldehyde and fumaric acid are also considered good candidates for biomarkers in serum samples (Table 2) [101,118,122].

In addition to the potential of a metabolic fingerprint in RA, metabolites in plasma have been linked to disease status, since acylcarnitine metabolites increase in lower disease activity and glucuronate, and hypoxanthine increased in higher disease activity [95]. As blood samples are beginning to be a powerful tool for metabolome analysis in RA patients, a study also provided a candidate biomarker panel with three metabolites in plasma samples, which may be a more filtrate technique in a metabolic profile for complement with transcriptome analysis (Table 2) [119].

### 3.4. Metabolites in Urine Samples of RA Patients

Since many metabolites are generated in trade-gastrointestinal processes during normal metabolism, urine and fecal samples are rich in metabolites from many signaling pathways in the human body. Urine, in particular, is an interesting sample source since its collection is very simple; it has a direct relationship with blood composition, which strongly supports the hypothesis that different molecular species are present in both biological fluids such as metabolites, nucleic acids or proteins and for which their variations are associated with pathological features [123]. Thus, blood and urine samples of RA patients presented similar disturbances in glycolysis, citrate cycle, oxidative stress, protein catabolism and urea cycle activity [104].

Twenty-eight significant associations have been reported between urine metabolite levels and disease diagnosis and three significant metabolite associations were reported with disease activity [124]. A cross-sectional study in urine samples showed that some metabolites from the TCA cycle such as citrate and fumarate were elevated in women, while carnitine, acetylcarnitine, acetone and creatinine were higher in men [125]. Moreover, lower levels of citrate in urine have been associated with inflammatory diseases such as RA [120,124].

In addition to a distinct urine metabolic profile that has been associated with RA pathogenesis, there are a few studies using urine samples, so it may be a potential target for a biomarker search. However, therapeutic drugs might also modify the circulating metabolomic profile and play a role in RA pathogenesis.

### 3.5. Metabolites as Predictors of Disease Activity

As discussed above, there are several metabolic pathways disrupted in inflammatory diseases; however, there is a lack of information about metabolism products as predictor or disease activity before/after different therapies [72]. The metabolic potential to predict outcomes was emphasized in a study that demonstrated that some endogenous metabolites may discriminate patients with regard to different disease activity [22] or even predict the response to a particular treatment [103]. Other studies attempted to use metabolomic technologies on patient-derived biospecimens for classifying patients with RA according to their disease activity categories [122,126,127]. Sasaki et al. (2019) identified metabolites in plasma and urine, respectively, that were differentially abundant between active RA (DAS28-ESR ≥ 3.2) and inactive RA (DAS28-ESR < 3.2) [128]. In addition, metabolites found in plasma were identified as circulating pro-/anti-inflammatory metabolic signatures that reflect disease activity and inflammatory status [128,129]. Ahn et al. (2020) reported 12 metabolites that may reflect disease activity and monitor joint damage [130]. These findings suggest that a wider application of metabolomic profiling—coupled with advanced analytics [61]—can lead to the discovery of novel and predictive biomarkers that complement current standard laboratory tests for assessing disease activity and therapeutics [107,131].

## 4. Therapeutics of RA and Metabolic Profile

The metabolic profile of RA patients changes considerably as the course of treatment is replaced, making the search for a biomarker even more complex [132]. The understanding of metabolic changes in RA may help to clarify disease pathogenesis, a very difficult task once therapeutics can be potentially driven by abnormal values or increasing anti-inflammatory metabolites. TNF is a potent pro-inflammatory cytokine that plays a key role in cellular metabolism, including glucose and lipid metabolism (Figure 3) [133]; therefore, changes in the metabolic profile are expected after the administration of its inhibitors [134]. 

The first study that evaluated changes in the metabolic profile in RA after treatment with TNFi (etanercept and infliximab) described increases in hippuric acid, citrate and lactic acid related to infliximab use, while increases in choline, phenylacetic acid, urea, creatine and methylamine were observed after treatment with etanercept [135]. TNF antagonist therapy also presented a link between urine metabolites and disease activity from responders and non-responders [136]; moreover, it was found a metabolite signature from carbohydrate derivatives, which may distinguish responsiveness from non-responsive patients using TNF inhibitors (Table 3) [134,137,138]. Rituximab administration, an anti-CD20 antibody, also seems to differentiate drug response by downregulated key lipids and amino acids in serum samples [134].

The different responsiveness according to the therapeutic is very often related to a change in the metabolite pattern. The metabolites of early RA patients were associated with sustained, drug-free remission (DAS28 < 2.6) after tocilizumab (TCZ)-or methotrexate (MTX) therapies [127]. A study using TCZ found that increased concentrations of 3-hydroxybutyrate and phenylalanine can improve the ability to predict TCZ responders [140]. In addition, TCZ treatment may modulate the arachidonic acid metabolism affecting the IL-6 signaling of this pathway [125]. Tryptophan, which is a substrate for the enzyme indoleamine-2,3-dioxygenase (IDO2), has been shown to be necessary for the activation of CD4+ T cells and autoantibodies production and may play a role in the development of mice models of arthritis [98]. Monitoring threonine and tryptophan serum levels allowed distinguishing RA patients depending on the therapy that was being used; for example, a decrease in tryptophan levels may be reversed by the addition of methotrexate (MTX) [139]. Artacho et al. (2020) reported associations of the gut microbiome and their genes with future clinical responses, including orthologs related to purine and MTX metabolism [144]. Thus, it has also been reported that RA patients treated with MTX presented changes in the plasma metabolic profile after the start of the treatment by bringing taurine, aspartate, alanine, lactic acid, adenosine and guanine back to normal levels [145].

Another major class of therapy currently used in RA patients is oral glucocorticoids (GC). GC inhibits phospholipase A, a key enzyme that hydrolyzes membrane phospholipids in inflammatory tissues. The effect of GC on phospholipase likely modifies the phospholipid profile and may be related to cardiovascular risks in RA [146]. Fu et al. analyzed GC therapy on serum polar lipids and observed an increase in lysophosphatidylcholines (LPC) and lysophosphatidylethanolamine (LPE) in female RA patients [143]. Dimethylarginine levels were lower in patients on chronic GC use compared with non-glucocorticoids users, suggesting that long-term GC treatments improved endothelial function and induce cardiovascular protective effects by modulating arginine metabolism [143].

In addition to monitoring responsiveness during treatment and clinical severity, metabolite changes have been also linked to pain. Pain, a dominant component of the patient-reported outcome, is present in nearly one-third of patients after 21 months of combination therapy [147]. Given the increasingly acknowledged implication of the JAK/STAT pathway and its blocking agents (tofacitinib/baricitinib) in the modulation of pain and nociceptive response, the JAK/STAT pathway and nociceptive cytokine signaling in rheumatoid arthritis and psoriatic arthritis are observed [147]. On this issue, tofacitinib and baracitinib, JAK inhibitors, demonstrated increased levels of omega-3 fatty acids and docosahexaenoic acid (DHA) in treated patients, which were associated with a significant decrease in pain [142].

## 5. Metabolites and Comorbidities Associated with RA

Extra-articular manifestations and comorbidities are frequently observed in RA, leading to increased morbidity and premature mortality. The most common comorbidities include CVD, gastrointestinal, renal and pulmonary disease [131]. CVD was already associated with elevated levels of plasma dimethylarginine and increased arginase activity as potential indicators of cardiovascular risk [148]. In interstitial lung disease (ILD) problems associated with RA were found where the serum levels of decanoic acid and morpholine were decreased, and glycerol increased [149]. Thus, metabolites analysis may clarify several extra-articular manifestations of RA.

Changes in body composition, such as reduced fat-free mass with stable or increased fat mass (FM) are usually observed in RA patients. Low muscle strength associated with a reduction in muscle mass and size is a clinical manifestation called sarcopenia and the reduction in lean mass with maintenance or increase in fat mass is a set of alterations is called rheumatoid cachexia (RC) [9]. In a recent systematic review with a meta-analysis conducted by our group, we estimated a prevalence of RC of 15–32%, and sarcopenia was reported to occur in 28–37% of patients with RA [7]. Both RC and sarcopenia have been associated with chronic inflammation and disease severity [8]. Unfortunately, a lack of precise assessment of skeletal muscle status and its changes over time potentially hinders proper diagnosis and treatment of sarcopenia and RC [7]. Several methods are currently used to estimate muscle mass, including anthropometry (e.g., body mass index), bioelectrical impedance analysis (BIA), imaging techniques (e.g., computed tomography), NMR imaging, dual-energy X-ray absorptiometry (DXA), ultrasound and, more recently, the D3–creatine dilution method [6]. However, the diagnostic performance of these methods is limited by high cost and low availability [129]. Thus, it is important to search for biomarkers related to skeletal muscle mass to provide the attending physician with viable options to predict the development, progression and staging of skeletal muscle wasting during routine follow-up of RA patients. Recently, specific metabolites have been implicated with muscle wasting and physical impairment in different pathological conditions, which may be a new field to explore simple and less invasive methods for the management of disease and comorbidities [13,150].

Metabolomic analysis based on NMR spectroscopy of biofluids may be used to assess skeletal muscle mass, and a urine-based approach would provide an easy, non-invasive collection method and a metabolite-rich source [150]. On this matter, higher fatigue scores in RA patients were associated with a metabolic pattern characterized by the downregulation of metabolites from the urea cycle, fatty acids, tocopherols, aromatic amino acids and hypoxanthine [148].

**Figure 4:**
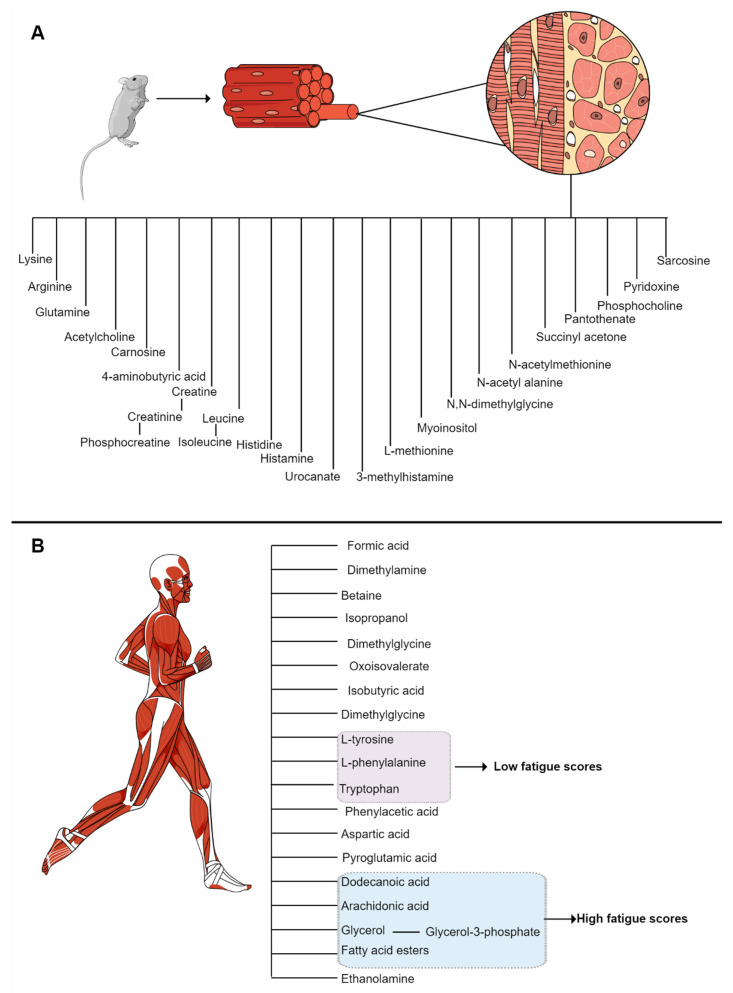
Metabolites associated with muscle wasting in RA. (**A**) Metabolites investigated during muscle wasting of RA mice models and (**B**) metabolites in RA patients associated with muscle loss and physical function.

There are a few studies that identified metabolites as biomarkers of skeletal muscle loss in RA patients. Our group has previously demonstrated that urine metabolomic profiles were associated with muscle wasting in the collagen-induced arthritis (CIA) model [150]. In this study, approximately 100 metabolites were identified and showed differences when comparing collagen-induced arthritis and control groups. In addition, twenty-eight metabolites were muscle-associated (Figure 4). In this sense, the findings identified by Alabarse et al. (2021) showed that muscle metabolic alterations may be detected in the urine of mice with CIA, and these results may allow greater validation in the urine of RA patients [150]. These findings suggest that these metabolites should be tested as biomarkers for the diagnosis, treatment and follow-up of muscle wasting related to RA.

## 6. Conclusions

Metabolomics is a powerful tool that can trace potential biomarkers and provide new insight into complex diseases such as AR. In the last decade, the metabolomics field has advanced toward better, more accurate analyses, such as targeted and untargeted approaches, and sample recovery by NMR methods, which make it possible the reuse of unique samples. Thus, metabolomics can be used to reveal disease-related changes before the onset of clinical findings, monitor disease activity and predict prognosis and drug responses. In this sense, metabolomics analyses of synovial fluid, blood and urine from RA patients have highlighted a set of metabolites as potential biomarkers. It is important to mention the relevance of TMAO and TCA cycle products in metabolic modulation during RA development. Moreover, a combined analysis with lipidomics search, especially cholesterol, FFAs and PUFAs, may provide important information before disease onset or preclinical disease, a stage in which several strategies for preventing full clinical disease development are currently being tested [1,81,82,83]. Synovial fluid analysis of lipids in RA patients opens alternatives to target key lipids as a therapeutic strategy without the immunosuppressive aspect of current treatments.

Several studies using TNF inhibitors observed tryptophan, valine, leucine, lysine, creatinine and alanine modulation, whereas JAK/STAT inhibitors may modulate exclusively fatty acids. Regarding MTX treatment, the gold standard for RA, the metabolic profile analysis was able to separate responders and non-responders during the course of treatment; the same was found in patients using TCZ and rituximab.

In terms of prognosis, little is known about metabolic modulation during a set of comorbidities, such as cardiovascular risk, lung interstitial disease or muscle wasting. There is an association between higher fatigue and an increase in urea cycle, fatty acids, tocopherols, aromatic amino acids and hypoxanthine in RA patients and, in the CIA model, muscle wasting was associated with a pattern of 100 metabolites present in the mice urine, indicating a potential for new biomarkers for this complication. As described, many studies have identified metabolites as biomarkers candidates for AR in several aspects of the disease, but there is a lack of evidence about disease-specific properties and the association with the metabolic profile. Only a few studies have combined omics approaches with current diagnostic tools, and validation studies are critically needed to confirm the identity and generality of putative biomarkers. Therefore, in the era of omics approaches, this new field of precise medicine is just beginning, and it has great potential of providing new evidence for the development and follow-up of RA and other autoimmune diseases.

## Figures and Tables

**Table 1:** Metabolites most investigated to establish a linking between disease and the metabolic profile.

Metabolite	Source	Mechanism of Action	Identified Condition	References
TMAO	Gut microbial metabolism from dietary choline and phosphatidylcholine (lecithin)	Increase glucose tolerance, inhibits hepatic insulin signaling and promotes adipose tissue inflammation	Increase in chronic kidney disease, type-2 diabetes mellitus, atherosclerosis	[32,33,34,35,36]
BCAA’s	Diet consumption of meat, dairy and vegetables	Induced NADPH inflammation and Akt/mTOR signaling, as well as promoting pro-inflammatory cytokines (IL-6, TNF) and blood of peripheral mononuclear cells by diet	Increase in: maple syrup urine disease, heart, kidney and spleen hypertrophy type 2 diabetes	[41,42,43]
Glutamine	Mainly synthesized by the GS and hydrolyzed by GLS.	Promotes enterocyte proliferation, regulates tight junction proteins, suppresses pro-inflammatory signaling pathways and protects cells against apoptosis and cellular stresses during normal and pathologic conditions	Trauma, sepsis, inflammatory bowel diseases and cardiovascular diseases	[44,45,46,47]
Succinate	TCA	Stabilizes transcription factor HIF-1a in tumors and in activated macrophages. Stimulates dendritic cells via its receptor succinate receptor1.	Peritonitis, cancer, diabetic and metabolic disease rodent models	[24,25,30,31]
Itaconate	TCA	Targets on ATF3-IκBζ pathway in a Nrf2-independent manner to mediate the inflammatory response.	Reperfusion injury, inflammatory disease and bacterial infections	[26,27,28,29]
Oxylipins	Oxygenation PUFAs: AA and LA	Activate PPARs or through GPCRs	Hyperlipidemia, hypertension, thrombosis, hemostasis and diabetes	[48,49,50]
SCFA	Products of dietary fiber metabolism by the gut microbiome	Activate FFA2 and FFA3 receptors and GPR109A through the inhibition of HDACs.	Salmonella infection, Eczema and Alzheimer’s disease	[51,52,53]
BA	BAs are synthesized in the liver and released into the gastrointestinal tract to aid in lipid digestion	Suppressed the production of LPS-induced inflammatory cytokines in macrophages	Insulin resistance	[54]
IDO	Tryptophan products	Toxic to T cells and induce cell death by apoptosis	Alzheimer’s disease, multiple sclerosis, Huntington’s disease and Human Lymphocyte Antigen-G	[40,55,56,57]
FFA	Derived from alpha-linolenic acid-omega-3-and linoleic acid-omega-6 or synthesized in the body.	Binding to cell-surface receptors of the GPCR family and regulated energy homeostasis indirectly via hormonal signaling	Type-2 Diabetes Colorectal cancer Systemic Lupus Erythematosus	[58,59]

Abbreviations: TMAO: choline and trimethylamine oxide; BCAA’S: branched-chain amino acids; TCA: tricarboxylic acid cycle; GLS: glutaminase; HIF-1a: hypoxia-inducible factor-1a; PUFAs: polyunsaturated fatty acids; AA: arachidonic acids; LA: linoleic acids; FFA: activate free fatty acid receptors; GPR109A: G-protein-coupled receptor 109A; HDACs: histone deacetylases; BA: bile acids; IDO: indoleamine 2,3-dioxygenase.

**Table 3:** Association of metabolites and the therapeutics of rheumatoid arthritis.

Source	Treatment Use	Method Applied	Metabolites	Reference
Urine	TNFi	GC/TOF MS	Histamine, glutamine, phenylacetic acid, xanthine, xanthurenic acid and creatinine were upregulated in urine samples from patients who had a good response to TNF therapy, while ethanolamine, hydroxyphenylpyruvic acid and phosphocreatine were downregulated.	[136]
Serum	DMARDS: MTX or leflunomide; bDMARDS: TNFi	HPLC-MS/MS	Threonine: Distinction of RA patients treated with MTX/leflunomide vs. infliximab/adalimumab/etanercept/tocilizumab and infliximab/adalimumab/etanercept/tocilizumab-prednisolone/NSAID Tryptophan: differentiated RA patients treated with methotrexate/leflunomide- vs. infliximab/adalimumab/etanercept/tocilizumab.	[139]
Serum	Etanercept	1H NMR	Increase in isoleucine, leucine, valine, alanine, glutamine, tyrosine and glucose levels and a decrease in 3-hydroxybutyrate levels N Etanercept good responders	[135]
Blood	Infliximab, abatacept or etanercept.	RP-UHPLC ESI-QTOF-MS	Two different metabolic profiles splitting good responders from non-responders: Carbohydrate derivatives (D-glucose, D-fructose, sucrose and maltose)	[137]
Plasma	Tocilizumab	H-NMR	Concentrations of 3-hydroxybutyrate and phenylalanine improved the ability to specifically predict TCZ responders	[140]
Serum	Rituximab	NMR-MS	Phosphatidylethanolamines, phosphatidyserines and phosphatidylglycerols were downregulated in responders; 37 lipids were different between responder and non-responders.	[141]
Serum	TNFi	CE-TOFMS	Association with TNFi: Betonicine, glycerol 3-phosphate, N-acetylalanine, hexanoic acid and taurine are associated with the response to TNFi in RA. Associated with Abatacept: Citric acid, quinic acid and 3-aminobutyric acid.	[138]
Serum	Tocilizumab	MS	Changes in arachidonic acid metabolism	[127]
Serum	Etanercept/adalimumab	1H NMR	3-hydroxyisobutyrate, lysine, L5, acetoacetate, creatine, GPC+APC, histidine and phenylalanine were elevated in RA, whereas leucine, acetate, betaine and formate were lower.	[134]
Serum	Tofacitinib/baricitinib	1H-NMR	Levels of omega-3 fatty acids DHA were increased in JAKi-treated patients. DHA was associated with decreases in pain.	[142]
Serum	GC	LC-MS/MS	Elevated lysophosphatidylcholines and lysophosphatidylethanolamines in women.	[143]

Abbreviations: GC/TOF MS: gas chromatography/time-of-flight mass spectrometry; NMR: nuclear magnetic resonance; LC-MS/MS: liquid chromatography tandem mass spectrometry; MTX: methotrexate; NSAID: TNFi: TNF-α inhibitors; CE-TOFMS: capillary electrophoresis-time-of-flight mass spectrometry; DHA: docosahexaenoic acid; JAK: Janus kinase inhibitors; UHPLC-HRMS: ultra-high performance liquid chromatography combined with high-resolution mass spectrometry. GC: glucocorticoids; DMARDS: disease-modifying antirheumatic drugs; b-DMARDS: biological disease-modifying antirheumatic drugs; RP-UHPLC: reverse-phase liquid chromatography–electrospray; ESI-QTOF-MS: electrospray QToF mass spectrometry.
